# Body and Mind

**Published:** 1876-03

**Authors:** 


*SECOND YEAR OF*

### BODY AND MIND

A SUPPLEMENT TO "HALL'S JOURNAL OF HEALTH," AND UNDER THE SAME MANAGEMENT AND EDITORSHIP. MONTHLY, 32 pages.

Subscription price only 75 cents a year, postage paid. Five copies one year, $3. Ten copies one year, $5. Single numbers seven cents. Postage on transient numbers, one cent each.

### BODY AND MIND

is the handsomest and the cheapest magazine of the kind in the world. It is intended to meet the demand for a Journal of Health and Miscellany, the low price of which will place it within the reach of all.

Every number is complete in itself, so that subscriptions may commence at any time. About six pages of each number are devoted to short articles on the care of the Health, and the remainder contains stories written expressly for it, and also carefully selected miscellaneous articles on subjects of interest to all intelligent people. The tone and influence of this publication will be kept up to the standard of "HALL'S JOURNAL OF HEALTH," so that it will be a welcome visitor to every family of education and refinement.

It will aim to give useful advice and instruction to all, but more especially to the young; teaching them to live so that their declining years may be years of joy, instead of sorrow and vain regrets over broken health. We invite all persons who feel an interest in the good work to act as our agents. Any one procuring 10 subscribers at 75 cents each, can retain 25 cents of each subscription. For each subscription over 10, 30 cents will be allowed.

A specimen number sent by mail, postage paid, on receipt of 5 cents. Twelve numbers sent, postage paid, for 50 cents. Owing to its great popularity and low price, and the liberal commissions we pay, there is no other publication on which Agents can do so well.

Any one who subscribes through us for any other publication costing $2 or more a year, will receive said publication at the subscription price during the time subscribed for, and BODY AND MIND during the same time FREE.

For example:

Any one sending us $2, which is the subscription price of the New York *Weekly Tribune,* will receive that paper by mail from the office of publication for one year, and BODY AND MIND for one year FREE. The same for any other Newspaper or Magazine in the United States, the subscription price of which is $2 a year, or over.

A limited number of advertisements will be received on reasonable terms. All P. O. money orders and drafts must be made payable, and all letters and papers addressed to the Editor of BODY AND MIND No. 137 Eighth Street, New York.

